# Short-Term Storability of Alginate-Encapsulated Persian Violet Microshoots for Germplasm Exchange

**DOI:** 10.3390/plants11020185

**Published:** 2022-01-11

**Authors:** Saowaros Phanomchai, Kitti Bodhipadma, Sompoch Noichinda, David W. M. Leung

**Affiliations:** 1 Division of Agro-Industrial Technology, Faculty of Applied Science, King Mongkut’s University of Technology North Bangkok, Bangsue, Bangkok 10800, Thailand; ph.saowaros@gmail.com (S.P.); kitti.b@sci.kmutnb.ac.th (K.B.); sompoch.n@sci.kmutnb.ac.th (S.N.); 2 School of Biological Sciences, University of Canterbury, Private Bag 4800, Christchurch 8140, New Zealand

**Keywords:** *Exacum affine*, encapsulation, in vitro conservation, synthetic seed, plant tissue culture

## Abstract

Microshoots have been widely used for micropropagation. It may be necessary to store microshoots for a short period of time, for example in germplasm exchange needing transport to other research groups. Here, we investigated the short-term storability of alginate-encapsulated Persian violet (*Exacum affine* Balf. f. ex Regel) microshoots at 4 °C and 25 °C. After storage, the encapsulated microshoots were sown on basal Murashige and Skoog medium for germination and viability determination using tetrazolium chloride staining. The results showed that one or five microshoots encapsulated with a single alginate layer could be stored at 4 °C for up to 30 days, while the percentages of germination and viability of the microshoots encapsulated with two layers of alginate were greatly reduced upon storage. This is the first report on the storability of alginate-encapsulated multiple microshoots, which could be a more efficient way to encapsulate microshoots used for short-term cold storage.

## 1. Introduction

Somatic embryos or non-embryonic propagules such as microshoots can be encapsulated using an alginate-based protocol to assist mass micropropagation of plants, particularly the sowing into an ex-vitro growing substrate [1,2,3,4]. If required, the encapsulated propagules or commonly called “synthetic seeds,” could possibly be used to facilitate short-term storage required for germplasm exchange or delayed sowing [5,6]. In most studies on synthetic seeds, a plant propagule is often enclosed within a single alginate coating [7,8], while variations of this approach, including double coating layers, have also been investigated [9,10,11,12].

Persian violet (*Exacum affine* Balf. f. ex Regel) with its eye-catching purple flowers is popular for decorative purposes in Thailand and is mainly propagated vegetatively because propagation by seeds reduces this plant’s fertility [13]. Micropropagation via microshoots encapsulated with a single alginate coating may be an additional tool to assist mass vegetative propagation of Persian violet [4,14] and short-term storage for germplasm exchange. It is, therefore, of interest to investigate the effect of alginate coating on the short-term storability of encapsulated Persian violet microshoots at 4 °C and 25 °C for up to 60 days. During an increased time in storage, there would be some humidity loss from the encapsulated microshoot, which would affect its viability and emergence out of the coating. A double-layer coating was hypothesized to better protect the microshoot during storage than a single-layer one because there would be more alginate hydrogel and a higher water content in the double coating.

Aromatic rice microshoots (size of 3–5 mm) encapsulated with a single alginate layer could be kept at 4 °C for 30 days [15], while 4–8 mm Begonia microshoot in single-layer encapsulation was able to be kept for up to 56 days [16], suggesting that a larger encapsulated structure could be kept for a longer time than a smaller one. We also investigated this possibility with encapsulated Persian violet microshoots. Since, in our previous study [14], it was found that Persian violet microshoots of the size of 2–3 mm were preferable for micropropagation, we compared the storability of a synthetic seed (a microshoot of 2–3 mm encapsulated with a single layer of alginate) and a larger encapsulated structure (termed herewith as synthetic fruit) with five microshoots each of 2–3 mm. The emergernce or ‘germination’ of a single microshoot from the artificial alginate coating is analogous to the embryo which emerges from a true seed and hence the encapsulated microshoot is termed herewith as a synthetic seed. In a naturally formed fruit there might be more than one seed and hence the term used herewith a ‘synthetic fruit’ for several microshoots encapsulated in alginate more resembles a true fruit.

In particular, we investigated the effects of the respective coating approaches on the emergence rate of the microshoots out of the alginate coating (‘germination of synthetic seeds and synthetic fruit,’ respectively) after storage at two different temperatures for various times.

## 2. Results and Discussion

### 2.1. Alginate Encapsulation

**Figure 1:**
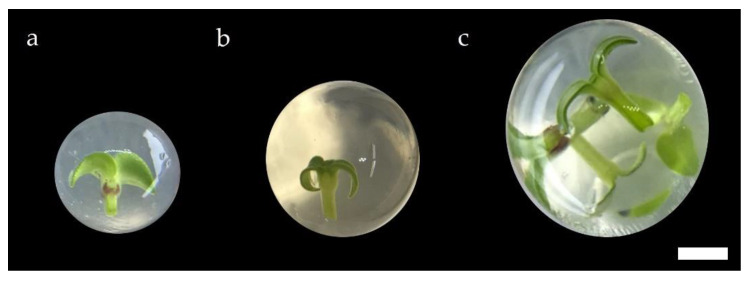
Appearance of alginate-encapsulated structures ((**a**): a Persian violet microshoot encapsulated with a single layer of alginate (a synthetic seed); (**b**): a microshoot encapsulated with a double layer of alginate (a synthetic seed); (**c**): five microshoots encapsulated within a single layer of alginate (a synthetic fruit); scale bar = 2 mm).

In this study, the diameters of the synthetic seed with a single and double alginate coating were about 5–6 mm and 7–8 mm, respectively (Figure 1). These were similar to those reported in other studies [12,17,18,19,20]. The diameter of the synthetic Persian violet fruit (10–11 mm) was higher than the synthetic seed. As far as we know, this is the first report of this variation of alginate encapsulation of microshoots or other propagules.

### 2.2. Germination and Viability Assessment

**Figure 2:**
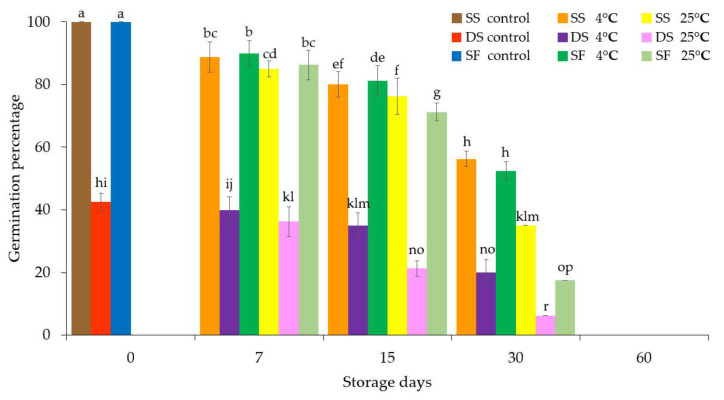
Germination percentages after synthetic seeds and fruit of Persian violet were stored at 4 °C and 25 °C for 0, 7, 15, 30 and 60 days. SS: a microshoot (synthetic seed) encapsulated with a single layer of alginate, DS: a single microshoot (synthetic seed) encapsulated with a double layer of alginate, SF: five microshoots encapsulated with a single layer of alginate (synthetic fruit). Data were means ± SD and those with different letters were significantly different (*p* < 0.05).

It was found that double alginate coating resulted in a substantially reduced germination rate (less than 50%) of Persian violet microshoots compared to a single coating prior to any storage treatment (Figure 2). After storage at 4 °C and 25 °C for up to 30 days, the microshoots encapsulated in double coating exhibited the lowest germination percentage compared to those in single coating encapsulating one or five microshoots (Figure S1 and Figure 2). Interestingly, there was no significant difference in the post-storage germination rates of the microshoots encapsulated individually in a single layer and the synthetic fruit. However, the apple and banana synthetic seeds encapsulated in a single alginate coating did not exhibit substantially different germination percentages than synthetic seeds with double alginate coating [11,12,17]. The effect of double coating on the storability of these synthetic seeds was also not the focus of these studies.

In general, after storage at 4 °C, all three encapsulated structures in the present study exhibited higher germination percentages than at 25 °C. It was also found that no encapsulated Persian violet microshoot was able to protrude out of the alginate coating after 60 days of storage at 4 °C and 25 °C (Figure 2 and Figure S2). This finding was similar to those of previous studies [21,22,23,24].

Different kinds of encapsulated propagules might respond differently to short-term cold storage. For example, the encapsulated somatic embryos of eggplant and *Catharanthus roseus* could be stored for 45, and 75 days, respectively [8,25], the encapsulated protocorms of *Cymbidium aloifolium* and the nodal buds of *Aquilaria malaccensis* could be stored for 90 days and 60 days, respectively [24,26]. The encapsulated microshoots of rice could be stored at 4 °C for 30 days [15], while those of Begonia and *Nerium oleander* could be stored at 4 °C for 56 days [16,27]. Overall, choosing the kind of propagule for encapsulation might be considered one of the factors for short-term low-temperature storage of plant germplasm in vitro.

**Figure 3:**
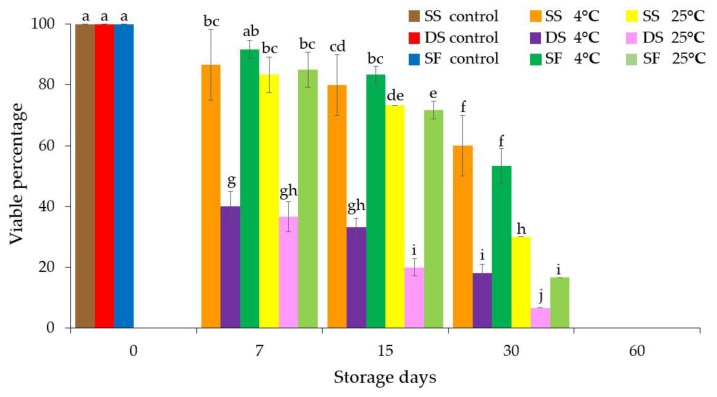
Percentages of viable Persian microshoots from synthetic seeds and fruit after storage at 4 °C and 25 °C for 0, 7, 15, 30 and 60 days. The viability test was based on staining the microshoots with 1% (*w*/*v*) 2,3,5-triphenyltetrazolium chloride. SS: a microshoot (synthetic seed) encapsulated with a single layer of alginate, DS: a single microshoot (synthetic seed) encapsulated with a double layer of alginate, SF: five microshoots encapsulated with a single layer of alginate (synthetic fruit). Data were means ± SD and those with different letters were significantly different (*p* < 0.05).

The percentages of viable microshoots in the synthetic Persian violet seeds or fruit were determined using tetrazolium tetrachloride staining decreased with increasing storage times at 4 °C and 25 °C (Figure 3 and Figure S3). This result was consistent with the finding of the germination percentages of synthetic seeds and fruit (Figure 2). However, double coating did not affect the viability of the encapsulated microshoots as revealed by tetrazolium tetrachloride viability staining. In this study, our present results will lead to future research of cryopreservation of the synthetic Persian violet seeds or fruit; for example, encapsulation–dehydration or encapsulation–vitrification, which has more benefit for long-term germplasm conservation [28,29,30].

## 3. Materials and Methods

### 3.1. Plant Materials

In vitro Persian violet plantlets were obtained and micropropagated as described previously [14]. Microshoots (2–3 mm long) were excised from the in vitro Persian violet plantlets and were then used for alginate encapsulation in this study.

### 3.2. Encapsulation of Microshoots

Persian violet microshoots were cut under aseptic conditions and then submerged in a 120 mL jar containing 25 mL of 3% (*w*/*v*) sodium alginate dissolved in basal liquid MS medium [31] supplemented with 3% sucrose. Using a 1000 μL micropipette (Mettler Toledo, Columbus, OH, USA) fitted with a micropipette tip (Axygen Scientific, Inc., Union City, CA, USA) that had been cut so that the inside diameter was 6 mm, five microshoots with the sodium alginate solution were drawn into the tip of the micropipette. The microshoots were then pushed out one by one in the case of generating synthetic seeds (Figure 1a,b), or the five microshoots were pushed out all at once in the case of generating synthetic fruit (Figure 1c), into a 240 mL jar containing 50 mL of 1% (*w*/*v*) calcium chloride dissolved in basal liquid MS medium supplemented with 3% sucrose. This procedure was repeated until 20 spherical synthetic seeds or synthetic fruit, each with a microshoot or five microshoots, respectively, were generated. The jar was shaken gently for 30 min before the synthetic seeds or synthetic fruit were rinsed with 50 mL of basal liquid MS medium supplemented with 3% sucrose in a 240 mL jar for 1 min. Some of the synthetic seeds with a single alginate layer were used to generate synthetic seeds with a double (second additional) layer of alginate (Figure 1b).

### 3.3. Storage Experiments and In Vitro Culture

During storage at 4 °C and 25 °C, 20 synthetic seeds or four synthetic fruit were kept in a 120 mL jar for 0, 7, 15, 30, and 60 days under aseptic, dark and controlled humidity conditions. After storage, a synthetic seed was transferred separately to a jar with basal semi-solid MS medium. For the synthetic fruit after storage, each microshoot with alginate coating was cut and transferred separately to a jar with basal semi-solid MS medium. All the synthetic seeds and microshoots from the synthetic fruit were cultured for 4 weeks in a growth room under light (20.87 μmol/m^2^/s) and dark periods (16/8 h, respectively) at 25 ± 2 °C. In this experiment, there were 4 replicates and 20 alginate-encapsulated structures in each replicate.

### 3.4. Germination and Viability Assessment of Synthetic Seed and Synthetic Fruit

At the end of in vitro culture, the number of microshoots that grew (‘germinated’) out of the alginate coating was counted to determine germination percentage according to Dewir et al. [32] using the following formula.

Germination percentage (GP) = (number of germinated microshoot from synthetic seeds and fruit/number of tested microshoots) × 100

For viability determination, each microshoot was removed from the coating and dissected slightly at the base to remove the attached calcium-alginate. Five microshoots were placed in a Petri dish containing 20 mL of 1% (*w*/*v*) 2,3,5-triphenyltetrazolium chloride for 24 h. A viable microshoot would turn red [33]. There were 3 replications and 10 synthetic seeds or synthetic fruit in each replication of the different storage treatments.

### 3.5. Statistical Analysis

The statistical software SPSS version 26 (IBM, Chicago, IL, USA), was used for data analysis. Differences of mean in the germination percentage and viability percentage of microshoots from the synthetic seeds and synthetic fruit of Persian violet were analyzed using Duncan’s Multiple Range Test (*p* < 0.05).

## 4. Conclusions

This research showed that storage of synthetic Persian violet seed and synthetic fruit with a single alginate coating is better at 4 °C than at 25 °C. If required, synthetic Persian violet seed and synthetic fruit with a single alginate coating could be stored at 4 °C for a short time (up to 30 days). Moreover, generating the synthetic fruit for cold storage would be a more efficient encapsulation procedure than encapsulating individual microshoots to generate synthetic seeds. The double alginate encapsulation approach is not suitable for cold storage of Persian violet microshoots.

## Data Availability

Data are contained within the article and Supplementary Materials.

## Supplementary Materials

The following are available online at https://www.mdpi.com/article/10.3390/plants11020185/s1, Figure S1: Characteristic of 4-week-old Persian violet plantlet grown out of encapsulated structure after storage at 4 °C for 7 days (a: single layer encapsulated synthetic seed; b: double layer encapsulated synthetic seed; c: synthetic fruit; scale bar = 1 cm).; Figure S2: Characteristic of non-viable Persian violet microshoots in synthetic fruit stored at 4 °C for 60 days (scale bar = 2 mm). Figure S3: Characteristic of Persian violet microshoot from synthetic fruit after stained with triphenyltetrazolium chloride (a: stored at 4 °C for 15 days; b: stored at 25 °C for 60 days; scale bar = 2 mm).
