# Supplementary material for: Short-Term Storability of Alginate-Encapsulated Persian Violet Microshoots for Germplasm Exchange
Source: Plants (Basel). 2022 Jan 11;11(2):185. doi: 10.3390/plants11020185 (PMC8779939; doi:10.3390/plants11020185)
Supplement: Supplementary file 1 [file plants-11-00185-s001.zip › plants-1532948-supplementary.pdf]

plants MDPI

Supplementary Figures

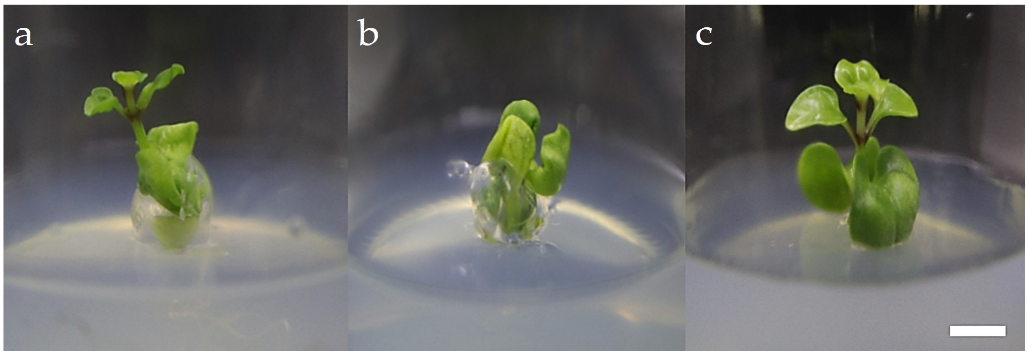

Figure S1. Characteristic of 4-week-old Persian violet plantlet grown out of encapsulated structure after storage at 4°C for 7 days (a: single layer encapsulated synthetic seed; b: double layer encapsulated synthetic seed; c: synthetic fruit; scale bar = 1 cm).

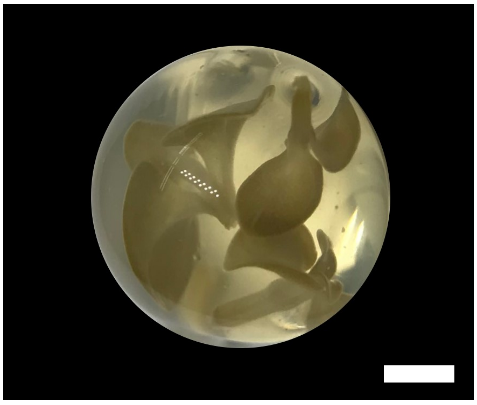

Figure S2. Characteristic of non-viable Persian violet microshoots in synthetic fruit stored at 4°C for 60 days (scale bar = 2 mm).

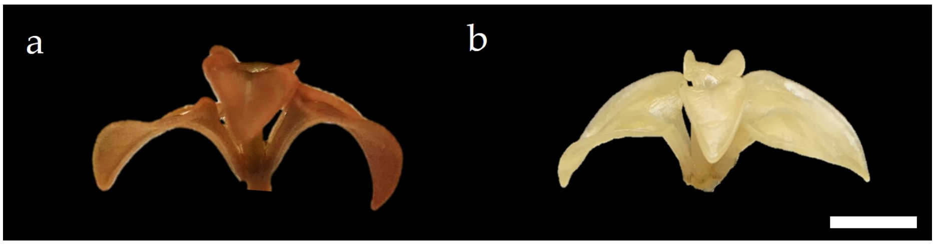

Figure S3. Characteristic of Persian violet microshoot from synthetic fruit after stained with triphenyltetrazolium chloride (a: stored at 4°C for 15 days; b: stored at 25°C for 60 days; scale bar = 2 mm).
